# Serum Spexin is Correlated with Lipoprotein(a) and Androgens in Female Adolescents

**DOI:** 10.3390/jcm8122103

**Published:** 2019-12-02

**Authors:** Flora Bacopoulou, Despoina Apostolaki, Aimilia Mantzou, Artemis Doulgeraki, Artur Pałasz, Pantelis Tsimaris, Eleni Koniari, Vasiliki Efthymiou

**Affiliations:** 1 Center for Adolescent Medicine and UNESCO Chair on Adolescent Health Care, First Department of Pediatrics, School of Medicine, National and Kapodistrian University of Athens, Aghia Sophia Children’s Hospital, 1 Thivon Street, Goudi, 115 27 Athens, Greece; ap_desp@yahoo.gr (D.A.); vikimiou2003@yahoo.gr (V.E.); 2 Unit of Clinical and Translational Research in Endocrinology, First Department of Pediatrics, School of Medicine, National and Kapodistrian University of Athens, 1 Thivon Street, Goudi, 115 27 Athens, Greece; amantzou@med.uoa.gr (A.M.); helenia8@yahoo.it (E.K.); 3 Department of Bone and Mineral Metabolism, Institute of Child Health, Aghia Sophia Children’s Hospital, 1 Thivon Street, Goudi, 115 27 Athens, Greece; doulgeraki@yahoo.com; 4 Department of Histology, School of Medicine in Katowice, Medical University of Silesia, ul. Medykow 18, 40-752 Katowice, Poland; apalasz@sum.edu.pl; 5 Division of Pediatric-Adolescent Gynecology and Reconstructive Surgery, Second Department of Obstetrics and Gynecology, National and Kapodistrian University of Athens, Aretaieio Hospital, 76 Vasilissis Sofias Avenue, 115 28 Athens, Greece; ptsimaris@gmail.com

**Keywords:** spexin, adolescents, lipoprotein(a), testosterone, free androgen index, DHEAS, androgens, fat mass, overweight, obesity

## Abstract

The *Spexin* gene is considered the most dysregulated in obese human fat. Limited data suggest that the novel peptide spexin may potentially impact food intake, weight regulation and body adiposity. The aim of this case-control study was to compare fasting serum spexin concentrations between normal weight (NW) and overweight/obese (OB/OW) adolescent females and explore the relationship between circulating spexin and anthropometric, bone and fat mass, metabolic and hormonal parameters. Eighty post-menarcheal females (mean age ± SD 16.23 ± 2.26 years); 55 NW (mean BMI ± SD 19.72 ± 2.52 kg/m^2^) and 25 OB/OW (mean BMI ± SD 29.35 ± 3.89 kg/m^2^) participated in the study. Circulating spexin levels did not differ significantly (*p* = 0.378) between NW (median (interquartile range), 0.26 (0.17) ng/mL) and OB/OW (median (interquartile range), 0.28 (0.06) ng/mL) adolescents and did not correlate with BMI (*r_s_* = −0.090, *p* = 0.438), % body fat (*r_s_* = −0.173, *p* = 0.409), glucose or insulin resistance indices derived from fasting and oral glucose tolerance states. In the total study sample, spexin concentrations correlated positively with lipoprotein(a) (*r_s_* = 0.402, *p* = 0.046). In the OB/OW adolescents spexin levels correlated positively with testosterone (*r_s_* = 0.727, *p* = 0.011) and free androgen index (*r_s_* = 0.755, *p* = 0.007). In the NW adolescents, spexin concentrations correlated negatively with dehydroepiandrosterone sulphate (*r_s_* = −0.445, *p* = 0.038). Results may suggest potential involvement of spexin in the regulation of lipoprotein(a) and of the reproductive/adrenal axis in post-menarcheal adolescent females.

## 1. Introduction

Spexin is a novel peptide which was first identified through a bioinformatics approach (Markov model) [1]. Immunohistochemical analysis in rats revealed its expression in a wide range of organs including the brain, where it may act as a novel multifunctional regulatory neuropeptide [2]. Subsequent studies with microarray techniques identified the *spexin* gene (Ch12: orf39) as the most dysregulated in obese human fat [3,4] with an almost complete absence of expression in obese human fat tissue in comparison to non-obese fat tissue.

Limited data from human and animal studies suggest that spexin may potentially impact food intake, weight regulation and body adiposity. In animal studies (rats with dietary-induced obesity and mice), spexin decreased caloric intake, increased locomotion and affected energy regulation with subsequent weight loss [4,5]. In vitro, spexin has also reduced adipocyte uptake of long-chain fatty acids [4]. Similarly, spexin functioned as a satiety factor through differential modulation of central orexigenic and anorexigenic signals in goldfish [6].

Data regarding circulating spexin in obese vs. normal-weight children are conflicting. Kumar et al. [7] observed a reverse association between body mass index (BMI) and serum spexin levels, with obese children having significantly lower circulating spexin than normal-weight children. Similarly, in the study of Chen et al. [8], serum spexin levels were significantly decreased in obese prepubertal children compared to controls and were negatively correlated with fasting insulin and the homeostatic model assessment for insulin resistance (HOMA-IR).

However, the proposed role of spexin in childhood obesity was questioned by Hodges et al. [9], who found that spexin did not correlate with any body composition, fitness or blood measurements.

In adults, circulating spexin has been associated with obesity, glucose metabolism and age. Recent research by Kolodziejski et al. [10] showed that serum spexin levels were reduced in obese women (vs. non-obese volunteers) and were negatively correlated with BMI, HOMA-IR and insulin. Lin et al. [11] reported that spexin levels were negatively correlated with BMI, fasting glucose and with age suggesting a possible role of this peptide in aging-related functions and disorders. Likewise, Gu et al. [12] reported lower circulating spexin levels in adults with type 2 diabetes mellitus (T2DM) as well as negative correlations of circulating spexin with blood glucose and hemoglobin A1c (HbA1c). Al-Daghri et al. [13] reported significantly lower spexin concentrations in adults (particularly women) with metabolic syndrome compared to those without metabolic syndrome. In another study, Al-Daghri et al. [14] demonstrated an inverse association between spexin levels with fasting glucose in adult females with prediabetes, but not in males, suggesting a sexual dimorphism in spexin levels, which according to the authors can be explained by gender differences in glucose metabolism and insulin sensitivity due to sex steroids [15].

Spexin may therefore have a role in metabolic regulation. The aim of this study was to compare fasting serum spexin concentrations between normal weight and overweight/obese adolescent females and explore the relationship between circulating spexin levels and anthropometric, bone and fat mass, metabolic and hormonal parameters.

## 2. Materials and Methods

### 2.1. Participants

Post-menarcheal adolescent females, aged 12–18 years, who presented to the Centre for Adolescent Medicine and UNESCO Chair on Adolescent Health Care of the First Department of Pediatrics at the Aghia Sophia Children’s Hospital, from May 2016 to June 2018, were eligible to participate in the study. Exclusion criteria included pregnancy, severe comorbidity and chronic medication or contraceptive use.

The study was approved by the Ethics Committee of the Aghia Sophia Children’s Hospital (project identification code 28126/09-12-15) and was in accordance with the Helsinki Declaration. Written consent was obtained from each adolescent and/or parent after full explanation of the purpose and nature of all procedures used.

### 2.2. Anthropometric Measurements

Weight (Wt), height (Ht), waist circumference (WC) and hip circumference (HC) were measured in all adolescents barefoot and in light clothing. Weight and height were measured by an electronic scale with stadiometer (seca 217, Hamburg, Germany). Waist and hip circumferences were measured with the use of an inextensible anthropometric tape (seca 201, Hamburg, Germany). Measurements were rounded to the nearest 0.1 cm.

### 2.3. Bone Mineral Density (BMD) and Body Fat Assessment

Study participants underwent a dual-energy X-ray absorptiometry (DXA) scan with enhanced pediatric software (General Electric Lunar Prodigy enCore 2008, Slough, UK) for assessment of bone and fat mass. Before scanning, a daily quality assessment was performed by the same radiologist using proper calibration phantoms, as per the manufacturer’s protocol. During the procedure, adolescents should wear light, metal-free clothing.

Bone status was evaluated by measurement of areal BMD (g/cm^2^) at two sites: the lumbar spine (LS, L1–L4) and the whole body without the head (WB). Measurement precision, as reflected by the coefficient of variation (CV), was 1.5% for lumbar spine BMD and 1.1% for subcranial whole body BMD. The results were presented as age and gender-matched BMD z-scores provided by the DXA manufacturer (pediatric Italian reference population). All patients with Ht <10th centile had their BMD z-scores corrected based on Ht-age, in accordance with pediatric guidelines for DXA interpretation [16].

For the purposes of this study and to highlight the possible association between circulating spexin levels and adiposity, body fat percentage (BF%) was also recorded and was automatically available from the WB scan.

### 2.4. Blood Sampling

Blood samples were collected from each participant in the morning between 08:00 and 09:00, after an overnight 8–10 h fast, between day 2 and day 5 of the menstrual cycle. Metabolic and hormonal parameters were analyzed immediately and the supernatant serum was kept frozen at −80 °C pending spexin assay. Participants were also evaluated with a 2-h oral glucose tolerance test (OGTT) for measurement of serum glucose and insulin concentrations at 0, 30, 60, 90 and 120 min. 

### 2.5. Measurements of Metabolic Parameters

Serum concentrations of glucose, triglycerides (TG), total cholesterol (CHOL), high-density lipoprotein cholesterol (HDL), low-density lipoprotein cholesterol (LDL) and alkaline phosphatase (ALP) were measured on an Architect Clinical Chemistry Analyzer (ABBOTT Diagnostics Ltd.). Lipoprotein(a) (Lp(a)) serum levels were measured using nephelometry, on the APTEC/KONELAB 60 automated analyzer. Apolipoprotein A1 (ApoA1), apolipoprotein B (ApoB) and apolipoprotein E (ApoE) were measured using latex particle-enhanced immunonephelometric assay on the BN PROSPECNEPHELOMETER (Siemens Healthcare Diagnostics, Liederbach, Germany). The intra-assay and inter-assay CV for ApoA1, ApoB, ApoE and Lp(a) did not exceed 5%. Serum interleukin 6 (IL-6) was measured using the BMS213HS ELISA kit from eBioscence Bender MedSystems GmbH. The method analytical sensitivity was 0.03 pg/mL, the calculated overall intra-assay CV was 4.9% and the overall inter-assay CV was 6.0%.

### 2.6. Measurements of Hormonal Parameters

Serum concentrations of follicle-stimulating hormone (FSH), luteinizing hormone (LH), estradiol (E2), testosterone (T), Δ4-androstenedione (Δ4-A), dehydroepiandrosterone sulphate (DHEA-S), sex hormone-binding globulin (SHBG), insulin, cortisol, intact parathyroid hormone (iPTH) and high sensitivity C-reactive protein (hsCRP) were measured on an Immulite 2000 analyzer (Siemens Healthcare Diagnostics Products Ltd., United Kingdom) using two-site chemiluminescent immunometric assays with analytical sensitivities for FSH 0.1 IU/L, LH 0.05 IU/L, E2 55.0 pmol/L, T 0.52 nmol/L, Δ4-A 1.0 nmol/L, DHEA-S 0.08 μmol/L SHBG 0.02 nmol/L, insulin 2 μIU/mL, cortisol 0.20 μg/dL, iPTH 3.00 ng/mL and hsCRP 0.1 mg/L. The intra-assay and inter-assay precision CVs ranged between 2.9–7.9% for FSH, 3.0–7.1% for LH, 6.7–16% for E2, 5.1–11.7% for T, 3.5–13.2% for Δ4-A, 4.9–13% for DHEA-S, 2.3–6.6% for SHBG, 3.3–7.3% for insulin, 5.2–9.4% for cortisol, 7.0–12% for iPTH and 2.8–8.7% for hsCRP. Serum 25-hydroxyvitamin D [25(OH)D] was measured on the MODYLAR ANALYTICS E170 (Roche Diagnostics GmbH) automated analyzer using electrochemiluminescence. Levels of serum 17-hydroxyprogesterone (17OHP) were determined by RIA (DIAsource ImmunoAssays SA, Belgium; intra-assay CV < 7%, inter-assay CV < 10%; analytical sensitivity 0.06 nmol/L). Serum free-testosterone (free-T) was measured by RIA using DSL (Diagnostic Systems Laboratories, Inc, USA) with analytical sensitivity 0.18 pg/mL. Free androgen index (FAI) was determined by the concentrations (in nmol/L) of T and SHBG with the use of the formula FAI = 100 × T/SHBG. Clinical hyperandrogenism was measured with the modified Ferriman-Gallwey (FG) score.

Serum spexin concentrations were measured by ELISA using the Spexin (Human) EIA Kit of Phoenix Pharmaceuticals (USA) with an analytical sensitivity of 0.08 ng/mL.

### 2.7. Insulin Sensitivity/Resistance Assessment

Insulin sensitivity was estimated with the use of the quantitative insulin sensitivity check index (QUICKI = 1/ [log (fasting insulin in μIU/mL) + log (fasting glucose in mg/dL)). Insulin resistance was estimated by the HOMA-IR according to the formula HOMA-IR = fasting glucose (in mg/dL) × fasting insulin (in μIU/mL)/405. By using 75 g OGTT, the total plasma glucose response and insulin secretion were evaluated from the area under the glucose curve (AUC glucose) and the insulin curve (AUC insulin), respectively, estimated by the trapezoid rule. The AUCglucose/AUCinsulin ratio was also calculated.

### 2.8. Ovarian Measurements

Study participants underwent transabdominal ultrasonography in the follicular phase of the menstrual cycle for measurement of ovarian volumes. The volume (V) of each ovary was calculated with the use of the formula V = length × width × height × 0.523. The mean ovarian volume (MOV) was calculated for each adolescent by using the mean volume of both ovaries.

### 2.9. Statistical Analysis

Continuous variables are shown as mean ± standard deviation (SD) or as median and interquartile range depending on data normality. Comparisons of continuous data were carried out with the use of the student *t*-test after checking the assumption of homogeneity of variance or with the Mann-Whitney U test for non-parametric data. Pearson or Spearman’s rho correlation coefficients identified correlations between continuous variables. BMI was calculated as the ratio of body weight to the square of height (kg/m^2^) and the International Obesity Task Force [17] cut-offs were used to categorize adolescents as normal weight, overweight and obese. P values were based on 2-tailed tests and statistical significance was set at *p* < 0.05. Statistical analysis was carried out using the SPSS software 25 version for Windows (IBM Corp. Released 2017. IBM SPSS Statistics for Windows, Version 25.0. Armonk, NY: IBM Corp.).

## 3. Results

A total of 296 girls were screened for eligibility but most families declined participation due to other commitments. Eventually 80 adolescent females (mean age ± SD 16.23 ± 2.26 years); 55 normal-weight (NW group; mean age ± SD 16.69 ± 2.22 years, mean BMI ± SD 19.72 ± 2.52 kg/m^2^) and 25 obese/overweight (OB/OW group; mean age ± SD 15.17 ± 2.01 years, mean BMI ± SD 29.35 ± 3.89 kg/m^2^), agreed to participate in the study. Participants’ anthropometric, bone and fat mass, metabolic and hormonal characteristics are presented in Table 1.

**Figure 1:**
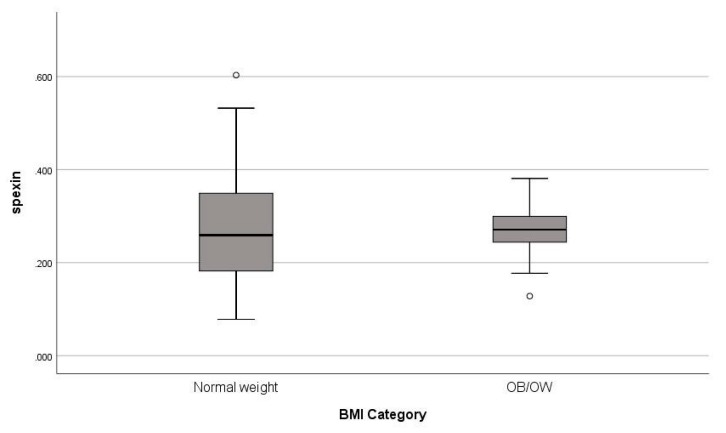
Differences in fasting serum spexin concentrations between normal weight and obese/overweight (OB/OW) female adolescents.

Statistically significant differences between the two groups were found in BMI, Wt, BF%, WC, HC, WC/HC, FG-score, glucose, AUC glucose, insulin, AUC insulin, AUC glucose/AUC insulin, HOMA-IR, QUICKI, T, SHBG, TG, CHOL, LDL, ApoB and LS z-score (Table 1). Circulating spexin levels did not differ significantly between NW and OB/OW adolescents (*p* = 0.378) (Table 1, Figure 1) and did not correlate with either BMI (*r_s_* = −0.090, *p* = 0.438) or BF% (*r_s_* = −0.173, *p* = 0.409) (Table 2).

In the total study sample, serum spexin concentrations were positively correlated with Lp(a) (*r_s_* = 0.402, *p* = 0.046). In the OB/OW group, serum spexin levels were positively correlated with T (*r_s_* = 0.727, *p* = 0.011) and FAI (*r_s_* = 0.755, *p* = 0.007). In the NW group, serum spexin concentrations were negatively correlated with DHEA-S (*r_s_* = −0.445, *p* = 0.038) (Table 2).

## 4. Discussion

In this case-control study, normal weight and obese/overweight adolescent females were compared in terms of fasting serum spexin concentrations. The relationship between the circulating spexin levels and anthropometric, bone and fat mass, metabolic and hormonal parameters was also explored.

The sample size was small, constituting a limitation of the study. However, the NW and OB/OW groups differed significantly in terms of WC, HC, WC/HC, glucose and insulin indices at the fasting state (glucose, insulin, HOMA-IR, QUICKI), and OGTT-based (AUC glucose, AUC insulin, AUC glucose/AUC insulin), T, SHBG, FG-score, lipids (TG, CHOL, LDL, ApoB), BF% and LS z-score. As expected, BMD was higher in OB/OW adolescents compared to NW, especially at the lumbar spine. According to the literature, increased body weight is typically correlated with increased BMD, without necessarily reflecting the quality of the bone, which can be more prone to fracture, given the metabolic bone-fat cross-talk [18].

Fasting circulating spexin concentrations did not differ significantly between NW and OB/OW post-menarcheal adolescents, neither did they correlate with BMI or BF%. Our findings also do not support the role of spexin in glucose homeostasis. Furthermore, we did not demonstrate any correlation between circulating spexin and the inflammatory cytokine IL-6, which is considered the best biomarker of obesity-induced, local and systemic low-grade inflammation associated with the development of insulin resistance [19,20].

Our results are in agreement with the findings of Hodges et al. [9] who examined the effect of obesity, T2DM and glucose administration on serum spexin concentrations in adolescents of both sexes. The median fasting spexin levels were similar between NW, OB or OB with T2DM adolescents with a mean age of 16 years. Moreover, they were not correlated with biochemical parameters (glucose, insulin, and lipids) or body composition and glucose ingestion did not affect serum spexin concentrations in these adolescents. In our study, serum spexin concentrations were not correlated with glucose or any of the surrogate markers of insulin resistance/sensitivity. In line with our findings, Kumar et al. [21] in a recent study of adolescents, did not find any correlation between spexin levels and fasting glucose or HOMA-IR. There are potential explanations for the lack of correlation between spexin and glucose metabolism and insulin sensitivity indices in our female adolescents, in contrast to adults. Insulin sensitivity and glucose metabolism fluctuate as the adolescent goes through various pubertal stages, and the factors influencing these changes have not been clearly defined. Early in puberty a decrease in insulin sensitivity occurs, which resolves by the end of puberty, possibly independently of changes in BMI or adiposity. On the other hand, a strong association between insulin resistance and BMI is evident in childhood [22].

Serum spexin concentrations were positively correlated with Lp(a) in the total study sample indicating that spexin may play a role in lipid metabolism. Recent data [23] advocate a novel role of spexin in lipid metabolism of human adipocytes by stimulating lipolysis and inhibiting lipogenesis. Gu et al. [12] in a study of adults with T2DM, found negative correlations for circulating spexin with blood lipids (LDL, TG). Lin et al. [11] reported that spexin levels were significantly correlated with TG. Serum spexin concentrations correlated negatively with TG in obese children in the study of Chen et al. [8].

Lp(a) is an atherogenic lipoprotein with high concentrations implicated in cardiovascular disease. It is composed of apolipoprotein B-100 and apolipoprotein(a), which shares extensive homology with plasminogen, conferring its unique atherogenic properties [24]. The variance of Lp(a) concentrations in largely explained by genetics; however, the physiological function of Lp(a) still remains unclear [25]. Studies in recent years have demonstrated unexpected inverse associations between Lp(a) concentrations and the risk of T2DM, i.e., an increased risk of T2DM in adults with low Lp(a) concentrations [26], as well as between Lp(a) levels and both fasting insulin and 2-h (post glucose challenge) insulin and glucose concentrations [27]. Furthermore, suppression of apolipoprotein(a) by insulin in hepatocytes has been demonstrated in experimental studies [28]. Likewise, serum spexin levels were down-regulated during glucose tolerance tests in adults with T2DM [12]. Although the relevance of the positive correlation between circulating Lp(a) and spexin is not known, as it is described for the first time, this association could be mediated by mechanisms implicated in glucose homeostasis.

In the absence of effective and safe drugs [29], regulation of lipoprotein and androgen status could contribute to atherogenic fraction reduction. In that context, spexin could be further evaluated in future studies as a promising target for novel therapeutic research. As expected, testosterone and SHBG concentrations were significantly lower in the OB/OW group (vs. the NW group). The inverse relationship between obesity and testosterone can be explained by the high levels of the enzyme aromatase in adipocytes, which converts testosterone to estradiol and may therefore lower circulating testosterone [30]. Serum concentrations of SHBG are also known to be negatively associated with obesity [31,32]. Interestingly, this study demonstrated for the first time an association between fasting serum spexin concentrations with Lp(a) and androgens.

More specifically, serum spexin concentrations correlated positively with T and FAI in the OB/OW group. In obese humans, spexin is the most down-regulated gene in their fat tissue [4] and serum spexin levels in severely obese (BMI > 35 kg/m^2^) adult women have been negatively correlated with obesity [10]. Obesity is also known to be correlated negatively with serum testosterone [30]. In the present study, BF% was significantly (*p* < 0.001) higher in the OB/OW group than in the NW group. Therefore, fat tissue may have played a role in the positive association between testosterone and spexin levels in our OB/OW group, although the inclusion of moderately (and not severely) obese adolescents in this group might not allow any differences relating to fat tissue level to become apparent in circulating spexin concentrations.

In the NW group, spexin concentrations correlated negatively with DHEA-S. The spexin gene belongs to the Spexin/Galanin/Kisspeptin gene family [33] suggesting its involvement in the regulation of reproduction. Spexin has multiple physiological functions with studies in goldfish revealing that spexin inhibits the reproductive axis [34]. Moreover, research on rat adrenal glands suggests that spexin inhibits the proliferation of adrenocortical cells, but the mechanism underlying this action remains unknown [35].

## 5. Conclusions

In conclusion, although spexin has been reported to be down-regulated in obese children and adults, serum spexin concentrations were not significantly different between the NW and OB/OW adolescents of our study. Also, our findings do not corroborate the proposed role of spexin as a biomarker of body adiposity and glucose control in the clinical setting, as circulating spexin levels did not correlate with fat mass, glucose or insulin resistance indices derived from fasting and OGTT states. The association of spexin with Lp(a) and androgens may suggest the potential involvement of spexin in the regulation of lipoprotein(a) and of the reproductive/adrenal axis in female post-menarcheal adolescents.

As spexin research is accumulating, further investigations are needed to explore the role of this evolutionarily conserved neuropeptide in humans.

## Figures and Tables

**Table 1:** Anthropometric, bone and fat mass, metabolic and hormonal characteristics of the study sample.

	Total Sample (*n* = 80)	NW Group (*n* = 55)	OB/OW Group (*n* = 25)	*p*
BMI (kg/m^2^)	22.72 ± 5.40	19.72 ± 2.52	29.35 ± 3.89	<0.001
Spexin (ng/mL) ^§^	0.27 (0.15)	0.26 (0.17)	0.28 (0.06)	0.378
BF (%)	36.43 ± 9.34	29.13 ± 4.56	44.33 ± 6.05	<0.001
Ht (cm)	163.62 ± 6.15	164.42 ± 5.48	162.75 ± 6.81	0.342
Wt (kg)	66.60 ± 16.34	55.50 ± 5.27	78.62 ± 15.77	<0.001
WC (cm) ^§^	77.00 (14.00)	69.00 (11.50)	84.00 (15.00)	<0.001
HC (cm)	103.39 ± 10.32	96.09 ± 5.63	107.05 ± 10.26	0.003
WC/HC ^§^	0.76 (0.09)	0.73 (0.07)	0.78 (0.09)	0.019
FG-score	8.47 ± 5.96	6.68 ± 4.85	11.29 ± 6.60	0.022
TG (mmol/L) ^§^	0.93 (0.53)	0.68 (0.44)	1.05 (0.45)	0.013
Glucose (mmol/L)	4.95 ± 0.51	4.75 ± 0.54	5.18 ± 0.37	0.002
AUC glucose (mmol/L)	793.03 ± 125.44	716.60 ± 94.28	838.88 ± 121.51	0.017
Insulin (pmol/L) ^§^	71.88 (65.63)	48.20 (41.04)	95.15 (87.99)	<0.001
AUC insulin ^§^ (pmol/L)	69,109.70 (56,754.54)	47,566.31 (12,885.41)	99,414.20 (57,473.35)	<0.001
AUC glucose/AUC insulin ^§^	1.40 (0.91)	1.82 (0.59)	0.92 (0.57)	0.002
HOMA-IR ^§^	2.19 (2.06)	1.36 (1.12)	3.26 (3.44)	<0.001
QUICKI	0.34 ± 0.03	0.36 ± 0.03	0.32 ± 0.02	<0.001
LH (IU/L) ^§^	5.40 (4.51)	5.68 (4.60)	4.62 (3.96)	0.130
FSH (IU/L)	5.27 ± 1.17	5.29 ± 1.13	5.25 ± 1.28	0.928
E2 (pmol/L)	148.05 ± 45.30	146.51 ± 47.94	150.51 ± 42.47	0.800
T (nmol/L)	6.66 ± 3.68	7.46 ± 4.13	5.03 ± 1.74	0.022
Free-T (pmol/L) ^§^	7.56 (7.08)	7.22 (8.54)	7.81 (10.27)	0.220
DHEA-S (μmol/L)	6.20 ± 3.16	5.54 ± 2.60	7.24 ± 3.76	0.118
Δ4-A (nmol/L)	11.10 ± 4.75	10.54 ± 5.34	11.97 ± 3.63	0.383
SHBG (nmol/L) ^§^	33.75 (22.88)	40.70 (23.58)	24.55 (13.15)	0.011
FAI ^§^	4.50 (5.20)	4.50 (6.95)	6.40 (3.60)	0.693
17OHP (nmol/L) ^§^	3.42 (2.30)	3.42 (2.51)	3.24 (1.39)	0.699
MOV (mL) ^§^	10.50 (6.38)	10.50 (8.00)	10.00 (5.11)	0.938
WB z-score	0.45 ± 0.91	0.06 ± 0.95	0.78 ± 0.77	0.065
LS z-score	0.36 ± 1.12	-0.19 ± 1.04	0.83 ± 0.99	0.030
ALP (IU/L) ^§^	83.00 (29.00)	86.00 (28.25)	80.00 (54.00)	0.892
iPTH (pmol/L)	5.32 ± 2.22	5.39 ± 1.64	5.27 ± 2.62	0.895
25(OH)D (nmol/L)	53.59 ± 19.02	57.58 ± 17.20	50.67 ± 20.32	0.370
Cortisol (nmol/L) ^§^	455.24 (193.13)	463.51 (126.09)	366.95 (466.55)	0.879
hsCRP (nmol/L) ^§^	11.62 (11.43)	8.95 (8.95)	13.43 (37.14)	0.190
IL-6 (ng/mL) ^§^	0.75 (0.66)	0.78 (0.78)	0.68 (0.63)	0.935
CHOL (nmol/L)	4.31 ± 0.87	3.69 ± 0.48	4.72 ± 0.82	0.001
HDL (nmol/L)	1.32 ± 0.25	1.39 ± 0.30	1.28 ± 0.21	0.240
LDL (nmol/L)	2.44 ± 0.76	1.91 ± 0.43	2.83 ± 0.73	0.001
ApoA1 (g/L)	1.33 ± 0.16	1.375 ± 0.18	1.31 ± 0.15	0.297
ApoB (g/L)	0.70 ± 0.20	0.58 ± 0.15	0.79 ± 0.18	0.004
ApoE (g/L)	0.04 ± 0.01	0.04 ± 0.02	0.04 ± 0.01	0.375
Lp(a) (g/L) ^§^	0.09 (0.19)	0.09 (0.16)	0.09 (0.19)	0.609
PRL (nmol/L) ^§^	4.79 (0.52)	0.67 (0.56)	0.42 (0.28)	0.097

NW: normal weight; OB/OW: obese/overweight; BMI: body mass index; BF: body fat; Ht: height; Wt: weight; WC: waist circumference; HC: hip circumference; FG-score: Ferriman–Gallwey score; TG: triglycerides; AUC: area under the curve; HOMA-IR: homeostatic model assessment for insulin resistance; QUICKI: quantitative insulin sensitivity check index; LH: luteinizing hormone; FSH: follicle stimulating hormone; E2: estradiol; T: testosterone; free-T: free-testosterone; DHEA-S: dehydroepiandrosterone sulfate; Δ4-A: Δ4-androstenedione; SHBG: sex hormone-binding globulin; FAI: free androgen index; 17OHP: 17-hydroxyprogesterone; MOV: mean ovarian volume; WB: whole body; LS: lumbar spine; ALP: alkaline phosphatase; iPTH: intact parathyroid hormone; 25 (OH)D: 25-hydroxyvitamin D; hsCRP: high sensitivity C-reactive protein; IL-6: interleukin 6; CHOL: cholesterol; HDL: high-density lipoprotein cholesterol; LDL: low-density lipoprotein cholesterol; ApoA1: apolipoprotein A1; ApoB: apolipoprotein B; ApoE: apolipoprotein E; Lp(a): lipoprotein(a); PRL: prolactin. Values are expressed as mean ± standard deviation (SD) or ^§^ median (interquartile range). The *p*-Value was calculated using the *t*-test after the assumption of homogeneity of variance or ^§^ Mann-Whitney U test.

**Table 2:** Spearman rho correlation coefficients between fasting serum spexin concentrations and study sample characteristics.

	Total Sample		OB/OW Group		NW Group	
	*r_s_*	*p*	*r_s_*	*p*	*r_s_*	*p*
Age (years)	−0.138	0.226	0.260	0.219	−0.215	0.115
BMI (kg/m^2^)	−0.090	0.438	−0.160	0.454	−0.253	0.067
BF (%)	−0.173	0.409	0.266	0.404	−0.135	0.661
Ht (cm)	0.077	0.596	0.190	0.374	−0.017	0.935
Wt (kg)	0.044	0.763	−0.041	0.848	0.026	0.901
WC (cm)	0.032	0.861	0.108	0.632	−0.123	0.719
HC (cm)	0.041	0.821	0.387	0.075	−0.543	0.085
WC/HC	0.027	0.879	−0.114	0.615	0.259	0.441
FG-score	0.092	0.594	0.333	0.245	−0.132	0.559
TG (mg/dL)	−0.232	0.174	−0.342	0.102	−0.042	0.897
Glucose (mg/dL)	0.039	0.789	−0.396	0.056	0.217	0.287
AUC glucose (mg/dL)	−0.118	0.582	0.181	0.519	−0.25	0.516
Insulin (μIU/mL)	0.102	0.483	−0.150	0.484	0.203	0.319
AUC insulin (μIU/mL)	0.213	0.317	0.309	0.262	0.017	0.966
AUC glucose/AUC insulin	−0.331	0.114	−0.342	0.212	−0.267	0.488
HOMA-IR	0.101	0.487	−0.221	0.300	0.218	0.284
QUICKI	−0.099	0.494	0.209	0.327	−0.213	0.296
LH (mIU/mL)	−0.056	0.744	0.503	0.067	−0.214	0.326
FSH (mIU/mL)	−0.034	0.843	0.068	0.817	−0.068	0.759
E2 (pg/mL)	0.018	0.919	0.314	0.274	−0.132	0.559
T (ng/mL)	−0.016	0.928	0.727 *	**0.011**	−0.147	0.504
Free-T (ng/dL)	0.080	0.662	0.222	0.446	−0.003	0.99
DHEA-S (μg/dL)	−0.212	0.216	0.143	0.626	−0.445 *	**0.038**
Δ4-A (ng/mL)	−0.285	0.093	−0.055	0.852	−0.38	0.081
SHBG (nmol/L)	−0.270	0.112	−0.471	0.089	−0.149	0.51
FAI	0.081	0.653	0.755 **	**0.007**	−0.105	0.642
17OHP (ng/mL)	−0.176	0.329	−0.007	0.983	−0.271	0.235
MOV (mL)	−0.079	0.643	−0.024	0.935	−0.087	0.693
WB z-score	−0.262	0.240	−0.319	0.313	−0.061	0.867
LS z-score	−0.124	0.582	−0.049	0.879	−0.040	0.913
ALP (IU/L)	−0.159	0.447	−0.456	0.088	0.225	0.532
iPTH (pg/mL)	−0.074	0.719	0.079	0.781	−0.387	0.239
25(OH)D (ng/mL)	−0.007	0.972	0.004	0.990	0.117	0.732
Cortisol (μg/dL)	−0.270	0.213	−0.308	0.306	−0.467	0.174
hsCRP (mg/L)	0.273	0.208	0.161	0.567	0.395	0.333
IL-6 (ng/mL) §	−0.085	0.687	0.307	0.265	−0.486	0.154
CHOL (mg/dL)	−0.011	0.953	−0.300	0.226	0.062	0.849
HDL (mg/dL)	−0.062	0.736	0.011	0.965	−0.161	0.616
LDL (mg/dL)	0.135	0.509	−0.089	0.752	0.288	0.391
ApoA1 (mg/dL)	−0.003	0.990	0.238	0.393	−0.315	0.345
ApoB (mg/dL)	0.056	0.787	−0.027	0.924	−0.014	0.968
ApoE (mg/dL)	0.104	0.619	−0.194	0.507	0.189	0.577
Lp(a) (mg/dL)	0.402 *	**0.046**	0.465	0.094	0.278	0.408
PRL (ng/mL)	−0.057	0.781	0.077	0.785	−0.182	0.592

NW: normal weight; OB/OW: obese/overweight; BMI: body mass index; Ht: height; Wt: weight; WC: waist circumference; HC: hip circumference; FG-score: Ferriman–Gallwey score; TG: triglycerides; AUC: area under the curve; HOMA-IR: homeostatic model assessment for insulin resistance; QUICKI: quantitative insulin sensitivity check index; LH: luteinizing hormone; FSH: follicle stimulating hormone; E2: estradiol; T: testosterone; free-T: free-testosterone; DHEA-S: dehydroepiandrosterone sulfate; Δ4-A: Δ4-androstenedione; SHBG: sex hormone-binding globulin; FAI: free androgen index; 17OHP: 17-hydroxyprogesterone; MOV: mean ovarian volume; WB: whole body; LS: lumbar spine; ALP: alkaline phosphatase; iPTH: intact parathyroid hormone; 25(OH)D: 25-hydroxyvitamin D; hsCRP: high sensitivity C-reactive protein; IL-6: interleukin 6; CHOL: cholesterol; HDL: high-density lipoprotein cholesterol; LDL: low-density lipoprotein cholesterol; ApoA1: apolipoprotein A1; ApoB: apolipoprotein B; ApoE: apolipoprotein E; Lp(a): lipoprotein(a); PRL: prolactin. §: median (interquartile range); *** *p* < 0.001, ** *p* < 0.01, * *p* < 0.05.
